# Supplementary material for: A Slow-Release Fertilizer of Urea Prepared via Melt Blending with Degradable Poly(lactic acid): Formulation and Release Mechanisms
Source: Polymers (Basel). 2021 Jun 3;13(11):1856. doi: 10.3390/polym13111856 (PMC8199746; doi:10.3390/polym13111856)
Supplement: Supplementary file 1 [file polymers-13-01856-s001.zip › polymers-1209890-SI.pdf]

A Slow-release Fertilizer of Urea Prepared via Melt Blending with Degradable Poly(lactic acid): Formulation and Release Mechanisms

Mujtahid Kaavessina, Sperisa Distantina and Esa Nur Shohih

Table S1. Urea concentration in water during the urea release test

Time, h	Concentration of Urea in Solution, g/100 ml				
	**SRF101**	**SRF201**	**SRF301**	**SRF203**	**SRF205**
0	0.0000	0.0000	0.0000	0.0000	0.0000
12	0.0029 ± 0.0002	0.0017 ± 0.0001	0.0019 ± 0.0002	0.0084 ± 0.0002	0.0168 ± 0.0002
24	0.0040 ± 0.0002	0.0028 ± 0.0002	0.0032 ± 0.0002	0.0129 ± 0.0003	0.0221 ± 0.0003
32	0.0045 ± 0.0002	0.0038 ± 0.0002	0.0039 ± 0.0002	0.0147 ± 0.0003	0.0241 ± 0.0003
48	0.0049 ± 0.0003	0.0049 ± 0.0002	0.0043 ± 0.0003	0.0167 ± 0.0002	0.0283 ± 0.0003
60	0.0054 ± 0.0002	0.0057 ± 0.0002	0.0047 ± 0.0002	0.0180 ± 0.0004	0.0309 ± 0.0003
72	0.0057 ± 0.0002	0.0066 ± 0.0003	0.0051 ± 0.0004	0.0187 ± 0.0001	0.0333 ± 0.0003
96	0.0063 ± 0.0003	0.0076 ± 0.0002	0.0057 ± 0.0003	0.0199 ± 0.0002	0.0359 ± 0.0004
120	0.0067 ± 0.0002	0.0081 ± 0.0002	0.0061 ± 0.0002	0.0219 ± 0.0003	0.0381 ± 0.0006
144	0.0071 ± 0.0003	0.0082 ± 0.0002	0.0066 ± 0.0003	0.0228 ± 0.0003	0.0399 ± 0.0004
168	0.0076 ± 0.0003	0.0083 ± 0.0001	0.0070 ± 0.0002	0.0257 ± 0.0002	0.0411 ± 0.0004
336	0.0085 ± 0.0002	0.0084 ± 0.0002	0.0078 ± 0.0003	0.0259 ± 0.0003	0.0437 ± 0.0003
504	0.0088 ± 0.0002	0.0086 ± 0.0002	0.0082 ± 0.0002	0.0262 ± 0.0002	0.0449 ± 0.0003

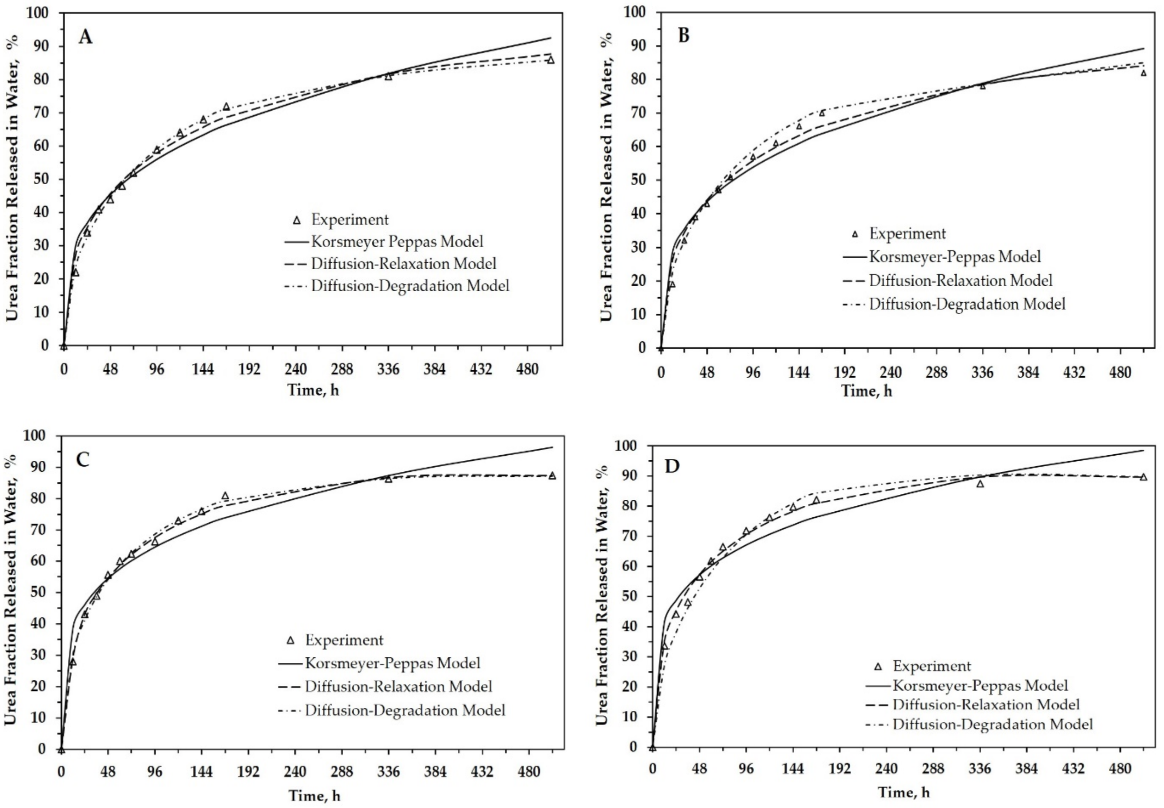

Figure S1. The urea fraction released in water as a function of time based on experimental and calculation data: (a) SRF 201, (b) SRF 301, (c) SRF 203, and (d) SRF 205
